# Targeting the apoptosis pathway to treat tumours of the paediatric nervous system

**DOI:** 10.1038/s41419-022-04900-y

**Published:** 2022-05-14

**Authors:** Marie-Claire Fitzgerald, Philip J. O’Halloran, Niamh M. C. Connolly, Brona M. Murphy

**Affiliations:** 1 Department of Physiology & Medical Physics, Royal College of Surgeons in Ireland, 31A York Street, Dublin, D02 YN77 Ireland; 2 National Children’s Research Centre at Children’s Health Ireland at Crumlin, Dublin, D12 N512 Ireland; 3 Department of Neurosurgery, Queen Elizabeth Hospital, Birmingham, UK; 4 Centre for Systems Medicine, Royal College of Surgeons in Ireland, 31A York Street, Dublin, D02 YN77 Ireland

**Keywords:** Cancer therapeutic resistance, Paediatric cancer, CNS cancer, Chemotherapy

## Abstract

New, more effective therapeutics are required for the treatment of paediatric cancers. Current treatment protocols of cytotoxic treatments including chemotherapy trigger cancer-cell death by engaging the apoptosis pathway, and chemotherapy efficacy is frequently impeded by apoptosis dysregulation. Apoptosis dysregulation, through genetic or epigenetic mechanisms, is a feature of many cancer types, and contributes to reduced treatment response, disease progression and ultimately treatment resistance. Novel approaches are required to overcome dysregulated apoptosis signalling, increase the efficacy of cancer treatment and improve patient outcomes. Here, we provide an insight into current knowledge of how the apoptosis pathway is dysregulated in paediatric nervous system tumours, with a focus on TRAIL receptors, the BCL-2 proteins and the IAP family, and highlight preclinical evidence demonstrating that pharmacological manipulation of the apoptosis pathway can restore apoptosis signalling and sensitise cancer cells to treatment. Finally, we discuss the potential clinical implications of these findings.

## Facts

- Apoptosis pathways are frequently dysregulated in cancer, including in paediatric nervous system malignancies and chemoresistance remains a significant clinical challenge
- Reactivation of apoptosis pathways represents a rational strategy to sensitise cancer cells to death-inducing treatments, and the development of targeted agents enables the pharmacological modulation of anti-apoptotic proteins
- A growing body of preclinical evidence highlights the utility of targeted agents such as BH3 mimetics and IAP inhibitors in paediatric oncology
- Techniques such as BH3 profiling will facilitate the clinical translation of some of these agents

## Open questions

- Can novel apoptosis modulators increase the sensitivity of paediatric nervous system cancers to apoptosis-inducing treatment?
- What biomarkers can identify patients likely to benefit from such treatments?
- Which combinations of targeted agents are most beneficial in these cancers?
- How can the apoptosis pathway be optimally targeted without resulting in on-target effects in non-disease tissue?

## Introduction

After accidents, cancer is the leading cause of death in children, as well as the leading cause of illness-related death [1, 2]. Between 2001 and 2010, ~400,000 children and adolescents were diagnosed with cancer worldwide [3]. Tumours of the nervous system are among the most commonly occurring paediatric cancers, with brain tumours the second most common solid malignancy [4]. Within malignant brain tumours, medulloblastoma is the most frequent, accounting for 20% of all cases [5]. Less common paediatric brain tumours include glioblastoma, astrocytoma, choroid plexus carcinoma, primitive neuro-ectodermal tumours (PNET), meningioma and ependymoma [6]. Tumours of the sympathetic nervous system such as neuroblastoma also occur frequently in young children, with 90% of neuroblastoma cases diagnosed in patients aged <5 years [7]. In the majority of these malignancies, chemotherapy is a mainstay of treatment [8–10], and is of particular importance in patients <3 years for whom radiation therapy is avoided due to its associated side effects [11].

**Fig. 1:**
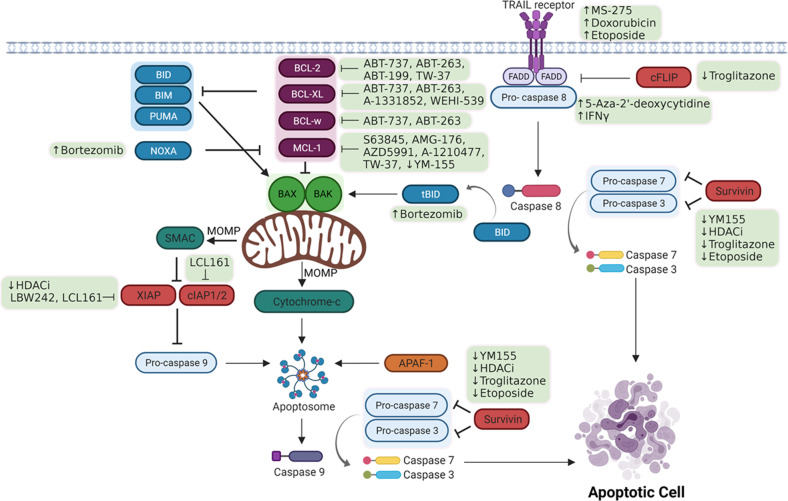
Schematic representation of the extrinsic and intrinsic apoptosis pathways, indicating pharmacological agents and their therapeutic targets. Proteins mediating similar functions are indicated by shading, while green shaded boxes highlight pharmacological agents. ↑ and ↓ indicates that the target is upregulated or downregulated by the drug, respectively. --| indicates inhibition of the target protein by the specified protein/drug. Created with BioRender.com.

Apoptosis is a tightly regulated form of programmed cell death carried out by multicellular organisms as part of normal development. The two main pathways of apoptosis are the intrinsic and extrinsic pathways, and these differ in their triggers and the means by which the apoptotic signal is transduced (Fig. 1). Both pathways converge on caspase activation, which leads to cell death execution [12]. Intrinsic apoptosis is initiated by alterations in the intracellular environment and is defined by mitochondrial outer membrane permeabilization (MOMP) [12]. The extrinsic pathway is triggered by activation of cell surface receptors by external signalling molecules or perturbations of the extracellular environment, and is propagated by caspase 8 activation [12].

Chemotherapy, and indeed most cancer therapies, target the apoptosis pathway to mediate their effects. Treatment success, however, is frequently hampered by apoptosis resistance. Indeed, evading apoptosis is one of the Hallmarks of Cancer, contributing to both tumour progression and treatment resistance [13]. Apoptosis resistance is either intrinsically present in cancer cells, for example through genetic mutations, or is acquired in response to cellular stress by upregulation or downregulation of anti- or pro-apoptotic proteins, respectively [13]. Characterising apoptosis defects can identify ways to restore apoptosis signalling, inform the development of novel anti-tumour strategies, and enable reduction of toxic chemotherapy regimens. This is of particular importance for paediatric tumours which are frequently associated with defective apoptotic machinery [14]. In this review, we focus on the ability of paediatric nervous system tumours to evade apoptosis, and highlight potential strategies to overcome the associated therapeutic resistance.

## Targeting the extrinsic apoptosis pathway in paediatric nervous system tumours

### The extrinsic apoptosis pathway

Extrinsic apoptosis is initiated by extracellular perturbations, generating a signal that is transduced by receptors located on the plasma membrane. Death receptors are activated by ligand binding. TRAIL, for instance, binds TRAIL-R1 and TRAIL-R2 (also known as DR4 and DR5) to initiate extrinsic apoptosis signalling [15]. In contrast, dependence receptors are activated when ligand levels drop below certain thresholds [16]. Death receptors are characterised by a cytoplasmic ‘death domain’ to relay the signal from the cell surface. TRAIL receptor ligation enables association of the FADD adapter molecule at the TRAIL-R1/R2 death domain [17], leading to formation of the death-inducing signalling complex (DISC) which recruits and activates caspase 8 [18]. Proteolytic activation of caspase 8 via interactions at the DISC initiates the caspase cascade and apoptosis execution. Cells are considered as type I or type II based on how they execute extrinsic apoptosis [19]. In type I cells, levels of activated caspase 8 are sufficient to drive apoptosis via activation of executioner caspases 3 and 7, and death is independent of the mitochondria [19]. In contrast, type II cells require involvement of the mitochondrial pathway to execute apoptosis, as DISC formation is lower in these cells [20]. Mitochondrial pathway involvement is mediated by caspase 8 truncation of BID, a pro-apoptotic protein [21], with truncated BID (tBID) subsequently engaging BAX and/or BAK to induce MOMP and the release of pro-apoptotic mediators, thereby amplifying the death signal (Fig. 1). Finally, cFLIP is a non-proteolytically active homologue of caspase 8 [22]. Its recruitment to the DISC forms an apoptosis inhibitory complex in conjunction with FADD [22], preventing caspase 8 activation. cFLIP therefore controls the susceptibility of tumour cells to TRAIL (Fig. 1).

### Targeting TRAIL receptors and downstream signalling

Exogenous TRAIL holds promise as a potential cancer therapeutic due to its selectivity for malignant cells with minimal toxicity against normal tissue [23], and its translational relevance has been increased by the development of new variants such as IZI1551 [24] and ABV-2661, a TRAIL-R agonist fusion protein [25] which is currently in a Phase 1 clinical trial for adult patients with previously-treated malignancies (NCT03082209).

The efficacy of TRAIL against cancer is limited, by frequent dysregulation of the extrinsic apoptosis pathway, including downregulation of TRAIL receptors and downstream signalling mediators, for example by epigenetic silencing [26]. Therefore, agents that restore expression of these key molecules have potential as TRAIL sensitisers, and the reversibility of epigenetic silencing is of clinical importance as epigenetic remodelling can be targeted pharmacologically.

Upregulation of TRAIL-R2/DR5 in neuroblastoma cells by etoposide [27, 28] or doxorubicin treatment [27] has been documented to increase TRAIL sensitivity. In medulloblastoma, TRAIL-R2/DR5 is commonly expressed, while TRAIL-R1/DR4 is frequently absent [29, 30]. This silencing of DR4 is understood to be mediated by aberrant histone deacetylation [30], and treatment of medulloblastoma cell lines with the histone deacetylase (HDAC) inhibitor MS275 increased DR4 expression, via increased acetylation of H3 and H4 at its transcriptional start site, and enhanced TRAIL-induced apoptosis [30].

Other potential strategies to sensitise cells to TRAIL treatment have been identified, including modulation of the BCL-2 protein family which is of particular relevance in type II cells [20]. The combination of TRAIL with the proteasome inhibitor Bortezomib was highlighted as an effective strategy for inducing apoptosis in cell line models and primary cultures of neuroblastoma [31] and meningioma [32]. Bortezomib enhanced TRAIL-induced, caspase 8-mediated tBID levels, and induced mitochondrial apoptosis and caspase activation through a mechanism involving p53 and upregulation of NOXA, a pro-apoptotic protein [31] whose role is described further in section 3.1. Encouragingly, this effect was also observed in an in vivo model of neuroblastoma [31], underscoring the potential clinical relevance of this combination. However, no clinical trials have been undertaken to assess the efficacy of this, as Bortezomib lacks the ability to cross the blood-brain barrier, limiting its relevance in the context of CNS tumours [33]. In this regard, another proteasome inhibitor, Marizomib, has increased potential as it can penetrate the blood-brain barrier, and is currently in a phase III trial in adult GBM patients (NCT03345095).

### Targeting caspase 8 to increase sensitivity to TRAIL

Weak caspase 8 expression is predictive of unfavourable progression-free survival (PFS) in medulloblastoma, where patients with low caspase 8 tumour levels had a 5-year PFS of 31%, compared with 73% in those with moderate or strong expression [34]. Additionally, loss or silencing of caspase 8 expression is associated with MYCN-amplified and aggressive neuroblastomas [35, 36].

Moreover, caspase 8 expression is key in determining TRAIL sensitivity, and loss of expression is frequent in the development of TRAIL resistance. Increased methylation of the caspase 8 promoter mediates its transcriptional silencing in medulloblastoma, PNET, and neuroblastoma [29, 34, 37–39]. Indeed, caspase 8 promoter methylation and subsequent silencing are associated with tumour aggressiveness in ganglioneuromas [39], and with invasiveness in TRAIL-resistant neuroblastoma cell lines [40, 41].

In this context, methylation inhibitors may be beneficial. In vitro treatment with 5-Aza-2’-deoxycytidine, a demethylating agent, successfully restored caspase 8 expression and sensitivity to TRAIL-induced apoptosis in cell line models of PNET [37], neuroblastoma [38, 40, 42] and medulloblastoma [42].

Furthermore, interferon-γ (IFNγ) treatment of caspase 8 deficient cells restored caspase 8 expression and TRAIL sensitivity in TRAIL-resistant medulloblastoma [34, 42] and neuroblastoma cell lines [27, 28, 43–46]. This was due to IFNγ-mediated regulation of an interferon-sensitive response element within the caspase 8 promoter [43, 47, 48]. IFNγ-mediated caspase 8 upregulation was also shown to enhance sensitivity of medulloblastoma cells to standard chemotherapeutic agents including cisplatin, doxorubicin, and etoposide, as well as to ionising radiation [49]. Together, these findings highlight that resistance to TRAIL-induced death may be overcome by targeting the epigenome to upregulate caspase 8 expression. The clinical importance of these findings is enhanced by the capability of 5-Aza-2’-deoxycytidine to readily penetrate the blood-brain barrier [50], and a phase I trial is underway investigating the safety of directly infusing this into the fourth ventricle in paediatric ependymoma patients (NCT02940483).

## Targeting the BCL-2 protein family in paediatric nervous system tumours

### The BCL-2 protein family

**Fig. 2:**
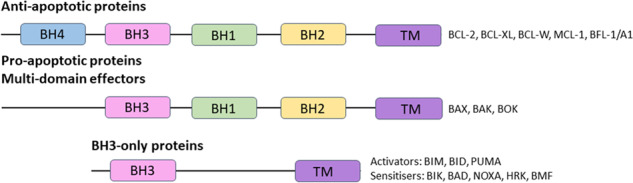
The structure of BCL-2 protein family members. TM denotes the transmembrane domain.

The intrinsic pathway of apoptosis is characterised by MOMP, which facilitates the cytosolic release of cytochrome c, SMAC, and other intermembrane space proteins, enabling apoptosome formation, caspase activation, and apoptosis execution. MOMP is tightly regulated by the BCL-2 protein family, whose members share homology in at least one BCL-2 homology (BH) domain (BH1, BH2, BH3 and BH4). On this basis they are classified into three groups, shown in Fig. 2: the anti-apoptotic proteins BCL-2, BCL-XL, MCL-1, BCL-W and BFL-1/A1 with sequence homology in all four BH regions, the pro-apoptotic proteins (termed multi-domain effectors) BAX, BAK and BOK with sequence homology at BH1, BH2 and BH3, and pro-apoptotic proteins such as BIM, BID, PUMA and NOXA, which only possess the short BH3 domain. This final group are designated as BH3-only proteins and can be further sub-classified as either ‘activators’ (BIM, BID and PUMA) or ‘sensitisers’, (BAD, BIK, BMF, HRK and NOXA) [51].

**Fig. 3:**
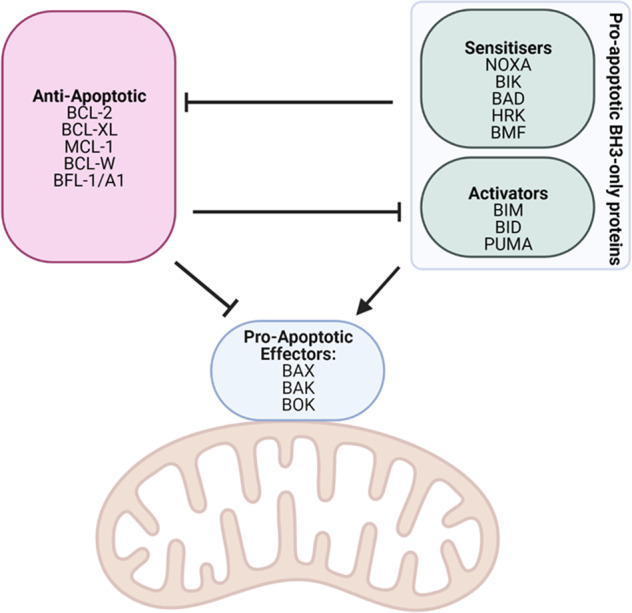
BCL-2 protein family interactions. The BCL-2 protein family interactions mediate BAX/BAK-induced mitochondrial outer membrane permeabilisation (MOMP). Created with BioRender.com.

BAX and BAK are MOMP effectors which dimerise via their exposed BH3 domains [52] and form higher-order structures through cross-linking of cysteine residues in their N-terminal epitopes [52]. These oligomers insert into the mitochondrial outer membrane lipid bilayer to form pores [53], leading to loss of mitochondrial membrane potential and disruption of mitochondrial structure, and culminating in release of cytochrome c and SMAC from the mitochondria [54]. While BOK is classified as a pro-apoptotic multi-domain effector protein, its role in apoptosis remains the subject of uncertainty and it is understood to be the least potent of the MOMP effector family [55]. As shown in Fig. 3, BAX/BAK activation is antagonised via direct interactions with the anti-apoptotic proteins BCL-XL, BCL-2, MCL-1, BCL-W and BFL-1/A1, which sequester pro-apoptotic proteins and prevent MOMP [51, 56–58]. Activator BH3-only proteins have the ability to bind non-activated BAX/BAK to directly trigger their activation [51]. Sensitiser BH3-only proteins mediate their pro-apoptotic functions indirectly, by binding anti-apoptotic proteins and competitively displacing BAX/BAK monomers or activator BH3-only proteins from the anti-apoptotic proteins [58, 59].

### BCL-2 proteins as therapeutic targets in cancer

Dysregulation of BCL-2 protein family members is reported in many cancers, and hence, their targeting may sensitise cancer cells towards apoptotic stimuli. For example, tumours frequently depend on an overabundance of anti-apoptotic signals to sustain their survival in the presence of death-inducing agents. In this regard, BH3 mimetics, a class of highly specific small molecule inhibitors that mimic the function of the pro-apoptotic BH3-only proteins, hold great potential.

ABT-737 was one of the earliest BH3 mimetics designed to inhibit the anti-apoptotic BCL-2 protein family. With nanomolar affinity for BCL-2, BCL-XL and BCL-W [60], ABT-737 demonstrated single-agent efficacy against lymphoma and small-cell lung cancer (SCLC), and synergy in combination with chemotherapy and radiation in vitro and in vivo. However, in vivo ABT-737 treatment resulted in platelet apoptosis [61] due to the exquisite anti-apoptotic dependence of platelets on BCL-XL [62], leading to reduced platelet and lymphocyte counts [60].

The clinical utility of ABT-737 was also hindered by its lack of oral bioavailability, prompting the development of ABT-263/Navitoclax, an orally available BH3 mimetic with a similar binding profile to ABT-737 [63]. Mechanistically, ABT-263 induces apoptosis by disrupting BIM:BCL-XL and BIM:BCL-2 interactions, releasing BIM and resulting in BAX-dependent MOMP [63]. ABT-263 demonstrated activity as a single agent in SCLC and haematological malignancies, resulted in tumour regression in SCLC and acute lymphoblastic leukaemia (ALL) xenograft models [63], and enhanced the activity of chemotherapeutic agents in vivo [63]. However, ABT-263 also induced platelet apoptosis and thrombocytopenia in vivo [61, 63]. Nevertheless, a Phase 1 study showed Navitoclax to be well tolerated in patients with SCLC and other solid malignancies [64], although a Phase II study showed limited efficacy as a single agent [65]. Results from the Paediatric Preclinical Testing Programme, which tested the efficacy of ABT-263 in a panel of 23 cell lines and 44 xenograft models, showed that ABT-263 had activity in approximately 50% of cell lines, but only limited single-agent activity in solid tumour xenograft models [66]. Further studies therefore focused on the effect of ABT-263 administered concurrently with other agents such as carboplatin and paclitaxel, though only modest anti-tumour effects were observed [67]. Nevertheless, a current clinical trial is evaluating its efficacy in combination with the MEK inhibitor trametinib in adult patients with advanced solid tumours (NCT02079740). It is possible that the identification of biomarkers of ABT-263 response could be used to identify patients likely to respond to ABT-263 treatment.

Specific inhibitors of individual anti-apoptotic proteins have also been developed, such as the BCL-2 inhibitor ABT-199/Venetoclax [68], BCL-XL inhibitors A1331852 [69] and WEHI-539 [70], and MCL-1 inhibitors S63845 [71], AMG-176 [72] and AZD5991 [73] (Fig. 1). These inhibitors have potential utility in the treatment of both paediatric and adult cancers, in blood and solid malignancies.

### BCL-2 proteins as therapeutic targets in paediatric tumours of the nervous system

With regards to paediatric tumours of the nervous system specifically, the anti-apoptotic BCL-2 protein family members are also potential therapeutic targets as their overexpression is commonly reported in these tumours, and correlates with clinical parameters such as therapeutic responsiveness and disease progression. BCL-2 expression in neuroblastoma [74–79] is associated with drug resistance, unfavourable histology and N-MYC amplification [74, 75], and a high-risk phenotype [76]. BCL-2 expression is also associated with poor outcome in paediatric glioblastoma [80] and ependymoma [81]. Interestingly, in medulloblastoma BCL-2 is the least frequently expressed of the anti-apoptotic BCL-2 proteins [82, 83], although expression is associated with poorly differentiated and highly proliferative tumour regions, and tends to correlate with poor outcome [84]. BCL-2 has also been identified as an important mediator of Hedgehog activity in the Sonic Hedgehog (SHH) subtype [85], and Gli1 and Gli2, essential transcriptional drivers of the SHH subtype of medulloblastoma, regulate BCL-2 transcription [86]. Indeed, mouse studies have shown that postnatal overexpression of BCL-2 promotes medulloblastoma tumour formation in cooperation with Sonic hedgehog pathway activation [87], highlighting a subtype-specific role for BCL-2 in medulloblastoma.

BCL-XL and MCL-1 are also of potential therapeutic interest. BCL-XL is the most frequently expressed anti-apoptotic BCL-2 protein in medulloblastoma tumours [83] and is expressed in the majority of medulloblastoma cell lines [88]. It is also expressed in most neuroblastoma cell lines [89], where its overexpression inhibits chemotherapy-induced apoptosis. Similarly, MCL-1 is also frequently expressed in medulloblastoma [~50% of tumours, [83]] and in neuroblastoma, where high expression is associated with a high-risk phenotype [76].

The identification of the anti-apoptotic BCL-2 proteins as therapeutic targets has led to the development of BH3 mimetics (Fig. 1), which have demonstrated efficacy in multiple cancer types in vitro as single agents or as sensitising agents. Early studies using the pan-BCL-2 inhibitor ABT-737 in neuroblastoma cell lines revealed a synergistic cytotoxicity with fenretinide, a synthetic retinoid derivative that inhibits cancer-cell line growth, while the combination also increased event-free survival in an orthotopic mouse model of neuroblastoma [90]. Subsequently, BCL-2 silencing in neuroblastoma cells was shown to induce apoptotic cell death [77] while ABT-199 also synergised with fenretinide in both neuroblastoma cell lines and PDX models expressing high BCL-2 levels, where improved event-free survival was demonstrated [91]. Mechanistically, neuroblastoma cell lines identified as BCL-2 dependent possess high levels of BCL-2 [92] and BIM:BCL-2 complexes, and ABT-199 mediates its effects by displacing BIM from BCL-2 [78, 79, 93], allowing mitochondrial apoptosis to proceed. MYCN-amplified neuroblastoma has been identified as particularly sensitive to ABT-199 treatment, and, in xenograft models, ABT-199 induced apoptosis and tumour regression in combination with the Aurora-A inhibitor MLN8237 [94]. In other xenograft models of BCL-2 dependent neuroblastoma [78], ABT-199 exhibited its effects through inhibition of tumour growth, rather than by inducing apoptosis. Results with ABT-263 [77] also suggested that ABT-263 mediates its effects in neuroblastoma through BCL-2 inhibition. Collectively, these studies identified a significant role for BCL-2 in mediating neuroblastoma resistance apoptosis, which could be overcome by utilising combinational treatment strategies.

Targeting MCL-1 or BCL-XL has also proven successful. Treatment with S63845 (MCL-1 inhibitor) or A1331852 (BCL-XL inhibitor) reduced viability and induced apoptosis in a subset of neuroblastoma cell lines, due to the release of pro-apoptotic proteins from BCL-XL- or MCL-1-mediated sequestration [92]. Furthermore, MCL-1 knockdown induced apoptosis and increased sensitivity to both etoposide and doxorubicin in cell line models of neuroblastoma [76], in a mechanism also understood to involve the release of BIM from MCL-1 sequestration [93]. The MCL-1 inhibitor A-1210477 is shown to synergise with the Hedgehog pathway inhibitor GANT61 in a subset of medulloblastoma cell lines [95]. BCL-XL targeting has additionally been demonstrated to sensitise medulloblastoma cells to MLN8237 treatment [96] as well as to ionising radiation [97], while synergy between ABT-263 and vincristine has also been attributed to its BCL-XL-targeting ability [98].

Combining BH3 mimetics has also emerged as a promising strategy to target BCL-2 protein-mediated resistance and enhance tumour cell killing, with early in vitro studies showing that MCL-1 knockdown overcomes ABT-737 resistance [76]. In some neuroblastoma mouse models, it was shown that ABT-199 treatment induced growth inhibition rather than apoptosis, due to MCL-1 upregulation and subsequent BIM sequestration by MCL-1 [78]. In vitro, MCL-1 inhibition sensitised neuroblastoma cells to ABT-199 treatment, highlighting the protective role MCL-1 mediates in cells with high BCL-2:BIM complex levels [78]. Furthermore, recent research demonstrated that dual inhibition of BCL-XL and MCL-1 synergistically reduces viability and induces death in neuroblastoma cell lines displaying treatment resistance to individual BCL-2, BCL-XL, or MCL-1 inhibitors [99]. Specifically, the efficacy of BCL-XL inhibition is limited by MCL-1, and the use of S63845 can therefore be used to abolish MCL-1-mediated resistance to A1331852 treatment by preventing MCL-1 binding pro-apoptotic the pro-apoptotic proteins displaced from BCL-XL by A1331852 [99]. The dual BCL-2/MCL-1 inhibitor TW-37 also successfully induces apoptosis in N-MYC-amplified neuroblastoma in vitro and in vivo [100], further emphasising that targeting multiple anti-apoptotic BCL-2 family members is necessary to induce apoptosis in some scenarios.

Clearly, the variable dependence of cancer cells on different anti-apoptotic proteins complicates the selection of the target protein. This is a particular concern in developing therapeutic combinations for paediatric patients, given that paediatric organs and tissues are more highly primed for apoptosis than the corresponding tissues of adults [101]. Fortunately, BH3 profiling represents a high-throughput experimental approach that can be used to elucidate dependencies of cancer-cell lines and patient tumour samples on individual anti-apoptotic proteins [102–104]. Studies in neuroblastoma, for instance, utilised BH3 profiling to predict responses to ABT-737 [105]. Alcon and colleagues have highlighted the potential of this approach in paediatric cancers [103], although further work is required to confirm the utility of BH3 profiling in the clinical paediatric cancer setting, and specifically in tumours of the paediatric nervous system.

Encouragingly, the lack of cancer-cell specificity of current BH3 mimetics is beginning to be addressed with the development of targeted inhibitors with a reduced toxicity profile. The dose-limiting platelet toxicity associated with BCL-XL inhibitors has led to the development of DT2216, a proteolysis-targeting chimera (PROTAC) that targets the BCL-XL protein for degradation via the Von Hippel-Lindau (VHL) (E3) ligase [106] in a cancer-cell specific manner. In vitro studies showed that DT2216 could sensitise prostate, TNBC, liver and colorectal cancer-cell lines to standard chemotherapeutic agents such as doxorubicin, docetaxel and vincristine [106], and this effect was also observed in a xenograft model of TNBC. Furthermore, DT2216 administered with ABT-199 completely suppressed tumour growth in a SCLC xenograft model [106], and also led to reduced disease burden and improved survival in a T-cell lymphoma PDX model [107]. DT2216 has entered a first-in-human clinical trial in patients with advanced/metastatic treatment-refractory solid or haematological malignancies (NCT04886622). A second compound selectively targeting BCL-XL in cancer is mirzotamab clezutoclax/ABBV-155 [108], an antibody-drug conjugate consisting of a BCL-XL inhibitor conjugated to a monoclonal anti-B7H3 antibody that targets the drug to cancer cells due to their selective B7H3 expression [109]. ABBV-155 is currently in dose-escalation studies in patients with relapsed or refractory solid tumours (NCT03595059). Trial results are eagerly awaited and potentially provide hope for the paediatric cancer community.

## Targeting IAP signalling in paediatric cancers of the nervous system

### The IAP family

**Fig. 4:**
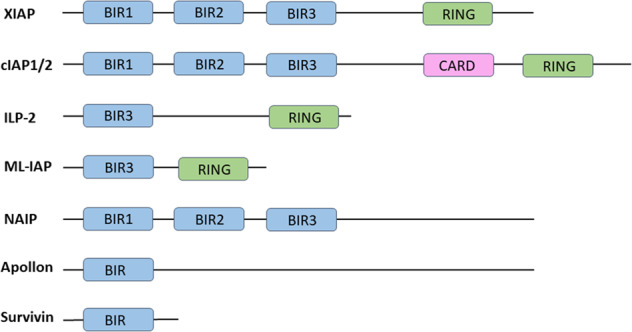
The structure of IAP family members. IAP members all have at least one baculoviral IAP repeat (BIR) domain that facilitates their interaction with other proteins. Some IAPs contain a Really Interesting New Gene (RING) domain that mediates E3 ligase activity. cIAP1/2 additionally contains a CAspase Recruitment Domain (CARD) which functions in protein-protein interactions.

The inhibitor of apoptosis (IAP) family of anti-apoptotic regulators prevent cell death by inhibiting caspases [110]. The IAP family has eight members, which are characterised by possession of at least one baculoviral IAP repeat (BIR) domain that mediates interactions with other proteins [111], as shown in Fig. 4. These are X-linked inhibitor of apoptosis protein (XIAP), cellular IAP 1 (cIAP1), cellular IAP 2 (cIAP2), IAP-like protein 2 (ILP-2), melanoma IAP (ML-IAP), neuronal apoptosis inhibitory protein (NAIP), Apollon and Survivin. XIAP is both the most potent and best studied member of the IAP family, and is the only member with the ability to directly inhibit activation of caspase 3, 7 and 9 [111]. The BIR2 domain has the ability to bind and inhibit caspase 3 and 7, while its BIR3 domain confers the ability to inhibit caspase 9 [112]. While cIAP1/2 and NAIP also possess all three BIR domains, they do not directly bind and inhibit caspases. cIAP1/2 indirectly inhibits caspase 3 and 7 by tagging them for proteasomal degradation, while NAIP constitutes part of the inflammasome, with roles in mediating innate immunity [113]. SMAC/DIABLO is an endogenous IAP antagonist which opposes the anti-apoptotic function of IAPs by interacting with and inhibiting their BIR2 and BIR3 domains via its N-terminal domain [114].

### The IAP family as therapeutic targets

IAP protein family members are dysregulated in both paediatric and adult malignancies. Medulloblastoma cell lines express higher IAP levels than normal astrocytes and brain tissue [115, 116], while XIAP is overexpressed in neuroblastoma cells compared with healthy adrenal gland tissue [117]. Additionally, cIAP1 and XIAP expression levels increase between primary and recurrent neuroblastoma in vitro, confirming their roles in aggressive recurrent disease [118]. Radiation and cisplatin treatment also upregulate protein and mRNA levels of cIAP1/2 and XIAP as a resistance mechanism [119].

IAPs, therefore, represent rational targets to directly sensitise cancer cells to apoptosis-inducing stimuli, and there are a diversity of pharmacological approaches capable of targeting IAPs. One potential mechanism is via SMAC mimetics which are based on the SMAC structure [120]. IAP suppression using SMAC mimetics effectively sensitises malignant cells to treatment with some classes of drugs in a cell line dependent manner [121]. For instance, treatment with the SMAC mimetic LCL161 activates both the extrinsic and intrinsic apoptosis pathways to overcome vincristine resistance in neuroblastoma cell lines [117, 118, 122]. Furthermore, LCL161 suppressed cell proliferation in medulloblastoma [116], and sensitised cells to both vincristine- and cisplatin-induced apoptosis with similar results observed with the IAP inhibitor, LBW242 [115, 116, 119]. Encouragingly, the combination of LBW242 and cisplatin was effective at inducing apoptosis in primary patient samples and a medulloblastoma xenograft model [119], as well as in CD133+ stem-like cancer cells [115] which are likely to be responsible for tumour initiation, maintenance and relapse [123]. Treatment with LCL161 was also shown to increase event-free survival in glioblastoma xenograft models [124]. While trials in xenograft models of paediatric cancers including neuroblastoma, medulloblastoma, ependymoma and glioblastoma have demonstrated limited activity of LCL161 as a single agent [124], combinatorial approaches may yet prove efficacious when tested in appropriate models.

Survivin is a member of the IAP family encoded by the *BIRC5* gene, and is an apoptosis suppressor expressed primarily in embryonal development, and upregulated in cancer [125]. In cell line models of neuroblastoma, Survivin expression is linked to high proliferation rates and resistance to drug-induced death [126]. Similar associations have been made clinically, where its expression tends to be associated with poor prognosis and high-risk disease [126, 127]. For example, gain of 17q25 which contains the Survivin locus occurs frequently in neuroblastoma [127, 128]. Indeed, gain of 17q25 is especially common in advanced neuroblastoma and correlates with worse outcome [127]. Survivin overexpression is also common in medulloblastoma [129], correlating with poor prognosis, lower survival and recurrence [129–133], and is predictive of poor clinical outcome independent of clinical staging [129]. High expression has been linked to various negative clinical features in a variety of nervous system cancers. Specifically, in choroid plexus carcinoma and ependymoma, high expression levels have been associated with increased tumour grade [134] and cell proliferation and poor patient outcome [135], respectively, while high expression has also been identified in paediatric glioma [136]. Therefore, Survivin expression has potential as both a prognostic marker and therapeutic target.

A number of approaches have been utilised to elucidate the effects of targeting Survivin. siRNA-mediated Survivin depletion decreased proliferation and promoted cell cycle arrest via accumulation of cells in G2/M [137], while disruption of the chromosomal passenger complex containing Survivin resulted in mitotic catastrophe and apoptotic death [127]. The miRNA miR-542-3p post-transcriptionally inhibits Survivin expression, and its expression correlates with better prognosis in neuroblastoma [138]. Xenograft models of neuroblastoma treated with miR-542-3p-loaded nanoparticles also had reduced Survivin expression, decreased proliferation and increased apoptosis [138].

YM155, also known as sepantronium bromide, suppresses Survivin expression by downregulating transcriptional activity at the Survivin promoter [139] and inhibits proliferation and promotes apoptosis in medulloblastoma [140], glioma [141, 142] and neuroblastoma cell lines in vitro [143, 144]. In xenograft models of neuroblastoma YM155 increases apoptosis and tumour regression, both independently and in combination with cisplatin [143]. Interestingly, however, cell lines that are sensitive to Survivin knockdown do not necessarily respond to YM155 [145]. A large-scale study using >100 neuroblastoma cell lines identified that ABCB1 or P-glycoprotein expression determines resistance to YM155 by their efflux pump activity [144, 145]. Consistent with this, ABCB1 inhibition using shRNA, cyclosporine or lapatinib sensitised cells to YM155 [145, 146]. YM155 and lapatanib treatment also decreased tumour size in an in vivo neuroblastoma model [146]. Additional evidence highlights that the anti-tumour effects of YM155 are not solely mediated by its modulation of Survivin, but also via its transcriptional downregulation of MCL-1 [141, 147, 148] and induction of DNA damage [148, 149]. In this context, YM155 has been shown to synergise with both BCL-2 family inhibitors ABT-737 [142] and ABT-263 [147] to induce apoptosis via MCL-1 downregulation. Furthermore, the combination of YM155 and TRAIL synergistically induced caspase-dependent cell death via downregulation of both MCL-1 and Survivin [141], highlighting how YM155 may effectively target multiple resistance mechanisms within cancer cells. A number of trials are in progress to evaluate the clinical activity of YM155 in adult solid and blood cancers. Results of these trials in combination with the encouraging preclinical results above could potentially lead to clinical trials in paediatric patients. Finally, troglitazone, an anti-hyperglycemic and anti-inflammatory drug used in the management of type 2 diabetes, has been shown to reduce expression of both cFLIP and Survivin, resulting in TRAIL sensitisation in neuroblastoma cells [150]. As troglitazone is commonly used in the clinic, it would be of great interest to assess its effects as a sensitiser in combination with other approved chemotherapeutic agents in the paediatric setting.

Lastly, HDAC inhibitors including NaB, SAHA and TSA have been shown to increase the sensitivity of neuroblastoma cells to TRAIL-induced death, through downregulation of XIAP and Survivin [36]. Currently, four HDAC inhibitors, vorinostat, belinostat, panobinostat, and romidepsin, are approved by the FDA for cancer treatment, with several others under clinical investigation. Of note, two clinical trials are ongoing to examine the activity of vorinostat and valproic acid in paediatric high-grade glioma in combination with temozolomide (NCT03243461, NCT01236560). Results from these trials could pave the way for trials in other paediatric malignancies.

## Conclusions

Tumours of the nervous system are among the most frequent malignancies in paediatric patients, and new treatment strategies are required to improve responsiveness and reduce the significant side effects associated with current treatment. Evasion of apoptosis is a Hallmark of cancer, with therapy resistance frequently associated with aberrant apoptosis signalling. A vast array of studies have characterised alterations in the apoptosis pathway that may contribute to defective apoptosis signalling, leading to the identification of druggable targets. The FDA approval of the BCL-2 inhibitor ABT-199/Venetoclax in 2016 was a critical development in the field of apoptosis-targeted therapy, highlighting the validity of this approach. The development of companion technologies such as BH3 profiling represents a means to personalise such therapeutic approaches.

Therapies targeting apoptosis have yet to translate to the paediatric setting, though many are under clinical trial investigation in adult patients. We here summarised the growing body of preclinical evidence that targeting the apoptosis pathway represents a promising strategy to mediate effective cancer-cell killing for the treatment of paediatric nervous system cancers. Current clinical trials in adults may open new avenues for more effective treatment of paediatric patients by identifying promising treatment combinations, while the development of specific cancer-targeting agents may further reduce the toxicity profile associated with current therapeutics and improve their potential clinical utility.
